# 

**Published:** 1890-09-06

**Authors:** 


### NOTICE TO CORRESPONDENTS.

All MS., Letters, Books for Review, and other matters intended for the Editor should be addressed The Editor, The Lodge, Porchester Square, London, W.

The Editor cannot undertake to return rejected M.S., even when accompanied by stamped directed envelope.

### Notice to Advertisers and Subscribers.

All Advertisements, Orders for Copies of The Hospital, and Business Communications should be addressed to The Publisher, *not to the Editor*, The Hospital, 140, Strand, W.C.

### Bound Volumes of "The Hospital."

Vol. VI., containing full page portraits of T.R.H. the Prince and Princess of Wales, and Miss Florence Nightingale, is obtainable at 4s., post free.

Orders will be received for Vol. VII., 4s. post free. Cases for binding may be had of the Publisher, at 1s. 6d. each.

### To Residents, Secretaries, and Matrons.

The Editor will be glad to have the Regulations and each Report as issued by Asylums, Hospitals, Convalescent and Nursing Institutions, as well as those of other Charities, and an early copy in duplicate of all Appeals and Festival Notices, etc., addressed to *The Editor, The Lodge, Porchester Square, London, W.* Any superintendent, secretary, matron, or other chief official who regularly sends such information may be registered, on application to the Publisher, to receive free of cost a cover for binding The Hospital in half-yearly volumes.

### BURDETT'S HOSPITAL ANNUAL FOR 1890

Is now ready, and may be obtained of the Publisher, The Hospital Offices, 140, Strand, W.C., price 2s. 6d. limp boards, or 3s. 6d. cloth. All orders must be accompanied by a remittance.

### General Charities.

[This Column is devoted to an account of work of charitable institutions other than Medical Charities. The Editor will be glad to receive reports or news of work at 140, Strand. *Philanthropy* should be plainly written on the envelope.]

**THE ROYAL NATIONAL LIFEBOAT INSTITUTION** at its meeting recently awarded the amount of £133 to the crews of its lifeboats and to shore boats, for bravery in saving or trying to save life from shipwreck. A new lifeboat has just been sent to St. Agnes (Scilly Islands).

The girls of the West Dulwich High School have been even more energetic than last year in collecting for the **CHILDREN'S COUNTRY HOLIDAYS FUND** (10, Buckingham Street, Strand). Hard as it seemed to beat their 1889 record, they have achieved it, and sent £150 to the treasurer.

Mr. John Odling, the secretary of **DR. BARNARDO'S HOMES**, writes to the *Times* to say that the expenses of the recent litigation in connection with this work have been defrayed entirely through sums collected for this specific object, and that not one shilling subscribed for the maintenance and rescue of the inmates has been used for it. We are very glad to see this notice, for it is wonderful how an erroneous idea is retained by the public.

**THE NAZARETH HOME OF REST**, Tandridge, Godstone, Surrey, has been opened for men and their wives, who can go there separately or together, or a mother may take a child provided it be under five years of age. The charge for men is 9s. a-week, and for women 8s. a-week. Sister Jane Francis will be glad of any contributions either pecuniary or in kind, games, books, &c.

**THE HOUSE OF CHARITY**, Greek Street, Soho, has for its main object the prevention of distress and misery, and this it tries to attain by affording relief after carefully investigating the character and antecedents of those who come for help. It finds situations, and gives a start to all sorts of persons who from no direct vice or idleness have come to destitution or want. Patients discharged from hospitals, orphans or friendless girls who have come up to London in search of employment widows who must work in order to live, emigrants, in fact people of any sort are afforded a gratuitous home, kindness and sympathy, and are in most cases found employment. Out of 554 cases received during the year, only five were dismissed for bad behaviour. The Rev. the Warden or the secretaries will be glad to give any further information about this charity.

There is no rescue home which has simpler methods of working than the **ANCHORAGE MISSION**, 32, Charing Cross. Those who wish to be admitted must be willing to conform to the regulations of a well ordered family. There is no hard and fast rule about how long the inmates may remain in the home. They are trained in household duties and found situations, and helped in every possible way to start afresh in life. The mission, which was started by Colonel Stuart Wortley in 1878, adds to its funds by needlework, and those who will help can do so by buying the articles of clothing made by the inmates. Mr. Arthur J. S. Maddison, the secretary, will gladly give further information.

"Music makes people milder and gentler, more moral and more reasonable." On this principle the **POPULAR MUSICAL UNION** started eight years ago to bring its influence to those of the London people whose poverty prevented their otherwise obtaining amusement and relaxation. The last year's work has included concerts and entertainments given in the poorest districts and the choral and orchestral classes given in different quarters, show by the large numbers who enter them that they are appreciated. Miss Foot is the new secretary, and her temporary address is 38, Wimpole-street, W. We are sorry to see that this society is in debt as it is doing excellent service, but the committee hope to be able to get over this difficulty shortly, and we recommend anybody who really cares for music to send a small subscription to the address we give.

**THE LONDON SOCIETY FOR TEACHING THE BLIND TO READ** sends us an annual report. It is a modest name to give to this society, for reading is only one of the many useful things it teaches. Writing, general information, music, piano tuning, knitting, basket work, sash-line making, and chair caning are all taught with a view to the future livelihood of the pupils. Some pupils are elected free of charge, and others can enter by payment. The society publish certain books in Lucas's embossed stenographic characters. Subscriptions may be sent to Captain Webber, 10, Upper Avenue Road, N.W.

Miss Harriett Little, Oaklands, Claughton, Cheshire, asks those interested in **WORK IN INDIA** to send her anything which will help to decorate the walls of 17 school houses of which she is superintendent in a native State in Western India. Large pictures such as the coloured prints of the different Christmas numbers or Christmas cards are much appreciated. This is easily understood; for the Mahrattee books used in the schools have scarcely any illustrations, generally only the portrait of some dismal-looking Hindoo God. If any of our readers could help her she would be very grateful; she leaves England on October 4th.

